# Feto-maternal outcomes of caesarean delivery in Federal Medical Centre, Asaba: a two year review

**DOI:** 10.4314/ahs.v22i1.22

**Published:** 2022-03

**Authors:** Sunday Jombo, Chukwuma Ossai, Daniel Onwusulu, Samuel Ilikannu, Adeniyi Fagbemi

**Affiliations:** 1 fmc, asaba, OBGYN; 2 Nnamdi Azikiwe University Teaching Hospital, OBGYN

**Keywords:** Caesarean section, caesarean section rate, maternal, fetal outcomes

## Abstract

**Background:**

The upward trend of caesarean section and its associated morbidity/mortality especially in low and middle income areas makes regular appraisal of the procedure necessary.

**Objective:**

To evaluate caesarean section; its rate, indications, and maternal and fetal outcomes in Asaba.

**Methods:**

A retrospective study of all caesarean sections carried out at the obstetrics unit of the Federal Medical Centre, Asaba, between July 1, 2018 and June 31, 2020. Data was analyzed using SPSS version 20.

**Results:**

There were 2778 deliveries during the period, out of which 705 had caesarean sections, giving an overall caesarean section rate of 25.4%.There were 456 (64.7%) emergency caesarean sections. The commonest indication for caesarean section was repeat caesarean section 196 (27.8%), while cephalo-pelvic disproportion 87 (12.3%) was the commonest indication for emergency caesarean section. Majority of the babies had low APGAR score at 1min and 5mins, 126 (27.6%) and 50 (11.0%) from emergency than elective caesarean section 16 (6.4%) and 5 (2.0%) at 1min and 5mins respectively (x2=17.963, P<0.001). There were 31 (4.2%) perinatal deaths out of which majority 28 (6.1%) were from emergency caesarean sections (x2=9.412 P=0.002). The commonest post-operative complication was postpartum anaemia (140 (19.9%) while caesarean section case fatality was 0.6%.

**Conclusion:**

This study showed a caesarean section rate of 25.4% with repeat caesarean section and Cephalopelvic disproportion being the most common indication for elective and emergency caesarean section respectively. Emergency caesarean section accounted for most of the cases and is associated with a higher risk of maternal and perinatal morbidity and mortality.

## Introduction

Caesarean section is the commonest operative delivery technique in the world. Caesarean section is the delivery of the fetus, membrane, and placenta through abdominal and uterine incision after age of fetal viability.^1^,^2^ The rate of caesarean section worldwide has been on the increase. The reasons include advanced maternal age due to the quest for higher education and career advancement thus placing them at higher risk of pregnancy complications. More so the use of electronic fetal monitoring and the universal adoption of the term breech trial. Others includes caesarean delivery on maternal request, decline in assisted vaginal birth and decline in the rate of vaginal birth after caesarean section.^3^ Also the safety of caesarean section as a mode of delivery has remrkably improved, due to increasing use of antibiotics, safe blood transfusion, and improved anesthetic techniques.^4^

The rate of Caesarean section is different across countries even between urban and rural areas, due to different socio-economic statuses, and access to public and private health care services.^5^ The first study on Caesarean section worldwide by Betrán et al., including 155 countries published from 1990 to 2014 reported 18.6% with a range from 6% to 27.2%. The rate was higher in developed countries and lower in developing countries. Latin America and the Caribbean region have the highest Caesarean section rate (40.5%), followed by Northern America (32.3%), Oceania (31.1%), Europe (25%), Asia (19.2%), and Africa (7.3%).^6^

In Nigeria, caesarean section rate varies from one region to the other but ranges from 9.9–35.5% as reported by different authors.^7^–^10^ In Enugu it was 25.7%^11^, Port-Harcourt, 30.3%^12^, Nnewi 18.5%8 and 34.5% in Eku Delta state.^13^

Caesarean section indications vary among different populations and countries, and there is no world standard classification system for indications of Caesarean sections.^14^ The most common indications for cesarean delivery include previous Caesarean section, multiple pregnancy, breech presentation, fetal distress, lack of progress in labor, small fetus and macrosomia, cord prolapse, transverse or oblique lie of the fetus, head and pelvis mismatch, previa or abruptio placenta, and severe pre-eclampsia.^15^

There are several documented adverse health outcomes associated with caesarean section both for the mother and their infants despite its relativel safety in recent times.^16^ In the neonate, caesarean section is associated with increased incidence of respiratory distress, high incidence of admission to the neonatal intensive care unit, prolonged hospitalization, low Apgar scores at birth, iatrogenic prematurity, birth traumas and transient tachypnea of the newborn.^17^ In the mother, it associated with higher maternal morbidity and mortality. Caesarean section is becoming increasingly used as a mode of delivery in Nigeria, and is a good practice to perform a periodic clinical audit of the fetal and maternal outcomes. This study aimed at determining the rate, indications and materno-fetal outcome of caesarean section in federal medical centre, Asaba.

## Specific objectives

- To determine the cesarean section rate in Federal Medical Centre, Asaba.
- To determine the common indications of cesarean section
- To compare the feto-maternal outcomes of cesarean section for both elective and emergency cesarean section.

## Materials and methods

### Study site

This study was carried out at the maternity complex of the Federal Medical Centre, Asaba. An average of 1,400 deliveries is conducted annually. It serves as referral center for both urban and rural population within and outside the state.

### Method

This was a two years retrospective study of all caesarean sections carried out at the Obstetric unit of the Federal Medical Centre, Asaba Nigeria, between July 1, 2018 and June 31, 2020. The data was retrieved from the theatre records, delivery register and case notes over the period under review and entered into a proforma created for this study.

The total number of deliveries during the period under review was also obtained from the annual reports of the department and labour ward. The proforma for each patient was checked for completion before it was entered into a spreadsheet. Data were collated and entered into the SPSS (IBM version 20) computer software.

### Statistical analysis

The Statistical package SPSS (IBM version 20) was used for data analysis. The results were expressed in frequencies, means, percentages, tables, figures. The Chi square test (χ2) was used for association at P = 0.05 at 95% confidence interval.

### Ethical consideration

Ethical clearance was obtained from Research and Ethics Committee of the Federal Medical Centre, Asaba.

## Results

There were 2778 deliveries during the study period, out of which were 705 cases of caesarean sections, giving an overall caesarean section rate of 25.4%. There were 456 (64.7%) of emergency caesarean sections and 249 (35.3%) elective caesarean sections. The age range of patients that had caesarean sections was between 16–50 years, with mean age of 31.81 ± 5.15 years. The age group with the most caesarean section was between 30–36 years 359 (50.9%), followed by 23–29 years 205 (29.1%). Almost all the patients 702(99.6%) were married. A greater proportion of the patients were Igbo 389 (55.2%), the least tribe being Itsekiri, 4 (0.6%) and Hausa 7 (1.0%). Majority of the patients 194 (27.5%) and 184 (26.1%) were civil servants and traders respectively, followed by housewife 101 (14.3%). Caesarean section was more common in women with tertiary level of education 530 (76.2%), followed by secondary level of education 156 (22.1%). The rate of caesarean birth was commonest in multiparous women 256 (36.3%) followed by nulliparous women 223 (31.6%) and Primiparous women 204 (28.9%). Five hundred and forty four (77.2%) of the women that had caesarean section were booked while the rest 161 (22.8%) where unbooked. Among the booked patients, 236 (94.8%) had elective caesarean section while 308 (67.5%) had emergency caesarean section. One hundred and forty eight (32.5%) of Unbooked patients had emergency caesarean section while 13 (5.2%) had elective caesarean section. The gestational age at which majority of the patients 486 (68.9%) had the caesarean section, was between 37–40weeks. 85 (12.1%) and 78 (11.1%) of the patients had the caesarean section between 34–36weeks + 6days and >40weeks respectively. Only 26 (3.7%) of the caesarean sections was done at <32weeks gestational age as a showed in table 1.

**Table 1:** socio-demographic characteristics

Variables	Frequency (N)	Percentage %
**AGE (Years)**		
16–22	24	3.4
23–29	205	29.1
30–36	359	50.9
37–43	108	15.3
44–50	9	1.3

**TOTAL**	**705**	**100**

**TRIBE**		
Igbo	389	55.2
Hausa	7	1.0
Yoruba	10	1.4
Urhobo	55	7.8
Isoko	30	4.3
Ukwuani	60	8.5
Itsekiri	4	0.6
Others	150	21.3

**TOTAL**	**705**	**100**

**MARITAL STATUS**		
Married	702	99.6
Single	2	0.3
Co-habiting	1	0.1

**TOTAL**	**705**	**100**

**EDUCATIONAL LEVEL**		
Primary	17	2.4
Secondary	156	22.1
Tertiary	530	75.2
Non-formal	2	0.3

**TOTAL**	**705**	**100**

**OCCUPATION**		
Civil servant	200	28.4
House wife	104	14.8
Farmer	4	0.6
Trader	211	29.9
Fashion Designer	38	5.4
Stylist	27	3.8
Applicant	10	1.4
Banker	12	1.7
Student	21	3.0
Teacher	44	6.2
Self employed	20	2.8
Corper	8	1.1
Lawyer	4	0.6
Pastor	2	0.3

**TOTAL**	**705**	**100**

**PARITY**		
Nulliparous	223	31.6
Primiparous	204	28.9
Multiparous	256	36.3
Grand-multiparous	22	3.1

**TOTAL**	**705**	**100**

**BOOKING STATUS**		
Booked	544	77.2
Unbooked	161	22.8

**TOTAL**	**705**	**100**

**GESTATIONAL AGE AT** **SURGERY (WEEKS)**		
<32	26	3.7
32–33 + 6	30	4.3
34–36 + 6	85	12.1
37–40	486	68.9
>40	78	11.1

**TOTAL**	**705**	**100**

The commonest indication for caesarean section was repeat caesarean section 196 (27.8%), followed by cephalopelvic disproportion 87 (12.3%), mal-presentation 70 (9.9%), severe pre-eclampsia / eclampsia 65 (9.2%), fetal distress 62 (8.8%), failed VBAC 57 (8.1%), placenta previa 34 (4.8%), abruption placenta with live baby 28 (4.0%). Multiple gestation 25 (3.5%), obstructed labour 21 (3.0%). Others include fetal macrosomia 15 (2.1%), severe oligohydraminious 11 (1.6%), cord prolapse 2 (0.3%) and others 32 (4.5%).

**Figure 1:**
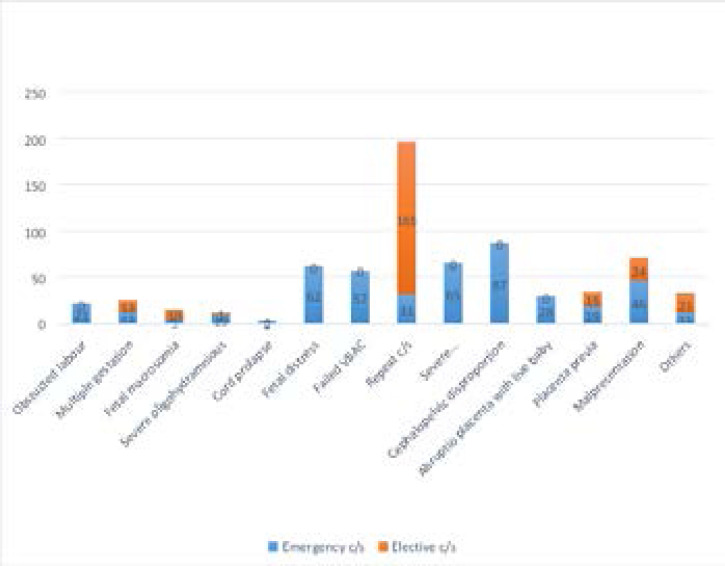
Indication for emergency / elective caesarean section

For elective caesarean section, the commonest indication was repeat caesarean section 165 (66.3%) followed by mal-presentation 24 (9.6%), while for emergency caesarean section, commonest indication was celphalopelvic disproportion 87 (19.1%) followed by severe pre-eclampsia / eclampsia 65 (14.3%) as showed in figure 1.

A total of 739 babies were delivered through caesarean section out of which 671 cases were singleton gestations and 34 were multiple gestations (Twin gestations). Majority of the babies were delivered through emergency procedure 474 (64.1%). The birth weights of most of the babies were normal birth weight 217 (87.1%) and 314 (68.9%) for elective and emergency caesarean section respectively. There were 17 (6.8%) macrosomic babies from elective caesarean section and 31 macrosomic babies from emergency caesarean section. More babies from emergency caesarean sections had extremely low birth weight, very low birth weight and low birth weight 7 (1.5%), 23 (5.0%) and 81 (17.8%) respectively than elective procedure 0 (0%), 1 (0.4%) and 14 (5.6%). (x2=38.79, p<0.001). Majority of the babies had low APGAR score at 1min and 5mins, 126 (27.6%) and 50 (11.0%) from emergency caesarean section than elective caesarean section 16 (6.4%) and 5 (2.0%) at 1min and 5mins respectively (x2=17.963, P<0.001). There were a total of 31 (4.2%) perinatal deaths out of which majority 28 (6.1%) were from emergency caesarean sections. (x2=9.412, P=0.002). There were more babies admitted into the neonatal unit from emergency caesarean section than elective caesarean section 134 (29.4%) and 17 (6.8%) respectively. (x2=46.53, P<0.001) as showed in table 2.

**Table 2:** Fetal outcome

	TYPE OF SURGERY					
Foetal outcome	Elective (N=705) n(%)	Emergency c/s (N=705) n(%)	Total (N=705) n(%)	X^2^	df	P value
**Birth weight**						
ELBW	0 (0)	7 (1.5)	7 (1.0)			
VLBW	1 (0.4)	23 (5.0)	24 (3.4)			
LBW	14 (5.6)	81 (5.0)	95 (13.5)	38.787	4	0.000*
NBW	217 (87.1)	314 (68.9)	531 (75.3)			
Macrosomia	17 (6.8)	31 (6.8)	48 (6.8)			
**Apgar Score at 1min**						
0–6	16 (6.4)	50 (11.0)	55 (7.8)	45.027	1	0.000*
7–10	233 (93.6)	330 (72.4)	650 (92.2)			
**Apgar Score at 5min**						
0–6	5 (2.0)	50 (11.0)	55 (7.8)	17.963	1	0.000*
7–10	244 (98.0)	406 (89.0)	650 (92.2)			
**Admission into** **Neonatal unit (NNU)**						
Yes	17 (6.8)	134 (29.4)	151 (21.4)	48.696	1	0.000*
No	232 (93.2)	322 (70.6)	554 (78.8)			
**Sex of babies**						
Male	151 (55.9)	240 (51.2)	391 (52.9)	17.963	1	0.000*
Female	119 (44.1)	229 (48.8)	348 (47.1)			
**Perinatal death**						
Yes	3 (1.2)	28 (6.1)	31 (4.4)	9.333	1	0.002*
No	246 (98.8)	428 (93.9)	674 (95.6)			

Total number of babies 739 (34 twins)

X^2^, chi-square, p-value < 0.05

* statistically significant.

Intra-operative blood loss greater than or equal to 1000mls (primary postpartum haemorrhage) was more in emergency caesarean section than elective caesarean section, 72 (15.8%) against 33 (13.3%) giving a total of 105 (14.9%) of primary post-partum haemorrhage (x2=0.817, P=0.366). The commonest post-operative complication was postpartum anaemia, 140 (19.9%). However, it was more in emergency caesarean section 95 (20.8%) than elective caesarean section 45 (18.1%). (x2=0.771, P=0.380). Other post-operative complications include puerperl sepsis, surgical site infection, caesarean hysterectomy and venous thromboembolism, which were more in emergency caesarean section. Prolonged hospital stay (≥ 7days) was more in emergency caesarean section 92 (20.2%) than elective caesarean section 32 (12.9%) (x2=5.96,P=0.015). There were four (4) cases of maternal death in the study period giving a case fatality rate of 0.6%, and all were from emergency caesarean section. Two of the cases resulted from massive intra-operative haemorrhage following major placenta previa with accreta while the other two were from complications of severe pre-eclampsia /eclampsia. (x2=2.197, P=0.138). All the four cases were unbooked as showed in table 3.

**Table 3:** Maternal outcome

OUTCOME	TYPE OF SURGERY					
	ELECTIVE (N=249) n(%)	EMERGENCY (N=456) n(%)	TOTAL (N=705) n(%)	X^2^	df	P value
**BLOOD LOSS (mililitres)**						
< 1000	216 (86.7)	384 (84.2)	600 (85.1)	0.817	1	0.366
≥ 1000	33 (13.3)	72 (15.8)	105 (14.9)			
**Post-partum anaemia**						
Yes	45 (18.1)	95 (20.8)	140 (19.9)	0.771	1	0.380
No	204 (81.9)	361 (79.2)	565 (80.1)			
**Duration of stay post-surgery** **(days)**						
< 7	217 (87.1)	364 (79.8)	581 (82.4)	5.960	1	0.015*
≥ 7	32 (12.9)	92 (20.2)	124 (17.6)			
**Wound dehiscience/breakdown**						
Yes	3 (1.2)	1 (0.2)	4 (0.6)			
No	246 (98.8)	455 (99.8)	701 (99.4)			
**Surgical site infection**						
Yes	2 (0.8)	4 (0.9)	6 (0.9)			
No	247 (99.2)	452 (99.1)	699 (99.1)			
**Puerperal sepsis**						
Yes	0 (0)	6 (1.3)	6 (0.9)			
No	249 (100)	450 (98.7)	699 (99.1)			
**Caesarean hysterectomy**						
Yes	0 (0)	2 (0.4)	2 (0.3)			
No	249 (100)	454 (99.6)	703 (99.7)			
**Icu admission**						
Yes	1 (0.4)	2 (0.4)	3 (0.4)			
No	248 (99.6)	454 (99.6)	702 (99.6)			
**Venous thromboembolism**						
Yes	0 (0)	1 (0.2)	1 (0.1)			
No	249 (100)	255 (99.8)	704 (99.9)			
**Maternal death**						
Yes	0 (0)	4 (0.9)	4 (0.6)	2.197	1	0.138
No	249 (100)	452 (99.1)	701 (99.4)			

X^2^, chi-square, p-value < 0.05

* statistically significant.

## Discussion

The overall caesarean section rate in this study was 25.4%. This rate was consistent with rates reported in Enugu (25.7%)^11^, and Ghana (26.9%).^18^ This rate was however higher than that reported in Abakiliki (16.4%)7, Nnewi (18.8%)8, Abuja (21.4%)^19^, Sokoto (11.3%)20, Obudu (18.1%)21 and world health organization (WHO) recommendation of 5–15%. But lower than the rate reported in Osogbo (35.9%)9 and Port Harcourt (30.3%).^12^

The high rate of caesarean section obtained in this study may be attributed to the fact that the study centre serves as a referral centre to most private and government owned health facilities in the area. These health facilities usually refer complicated obstetric emergencies to the study centre. A significant number of these referred cases were offered emergency caesarean section as a lifesaving intervention, hence the high caesarean section rate recorded. Other reasons may be due to the decreasing number of VBAC, decreasing skills for operative vaginal deliveries and presentation as well as increasing fear of litigation. However, caesarean section rate of 25.4% for a tertiary centre like federal medical centre, (FMC) Asaba may not be considered as abnormally high based on the recent WHO statement on caesarean section. WHO states that caesarean section should be undertaken when medically indicated and deemed necessary. It states that rather than striving to achieve a specific caesarean section rate effort should focus on providing caesarean section to all woman in need, and it is the responsibility of the health care provider to determine the woman that needcaesarean section on case by case basis.^22^,^23^

Emergency caesarean delivery contributes 64.7% of all caesarean section in this study compared to elective caesarean section 35.3%. This finding was similar in other studies in Kano, Sokoto, Obudu, Abuja, where emergency caesarean section constituted majority of the caesarean section.^10^,^19^,^21^,^24^ This is due to various indications during labour and partly because some of these patients come to the hospital as referral from other centers. Of the patients who had emergency caesarean section, 32.5% were unbooked.

The commonest indication for caesarean section in this study was repeat caesarean section followed by caphalopelvic disproportion. This compares similarly with studies in Enugu, Osogbo, Ghana, Nnewi and in the developed world where approximately 30% of caesarean deliveries were as a result of repeat caesarean sections. 8,9,18,25, 26 This may be attributed to the increasing rate of primary caesarean section and the decreasing trial of vaginal delivery.

The commonest indication for emergency caesarean section was cephalopelvic disproportion 19.1% (87/456) followed by severe pre-eclampsia /eclampsia 14.3% (65/456) and fetal distress 13.6% (62/456). This agrees with the study in Obudu and Port-harcourt where cephalopelvic disproportion was the most common indication for emergency caesarean section.^12^, ^21^ Majority of the babies had normal birth weight 217 (87.1%) and 314 (68.9%) for elective and emergency caesarean section respectively. However, there were more babies with extremely low birth weight, very low birth weight and low birth weight in emergency caesarean section than elective caesarean section (x2=38.79, df=4, p < 0.001). This may be due to the fact that the second commonest indication for emergency caesarean was severe pre-eclampsia /eclampsia with unfavorable cevix and majority of their cases occurred preterm. There were more cases of poor APGAR score (At both first and fifth minute), among babies from emergency c/s than elective c/s (x^2^=45.027, df=1 p<0.001 and x^2^=17.963, df=1, p<0.001) “need for admission into the neonatal unit and perinatal death” (x^2^=46.53, df=1, p<0.001, x2=9.419, df=1, p=0.002). This trend was similar in the study done in Obudu and Sokoto.^20^,^21^.

The maternal outcome was generally better for elective caesarean section than emergency caesarean section. Post-partum anaemia was the major complication in this study and occurred more in emergency caesarean section than elective although not statistically significant (x^2^ = 0.771, df =1, p =0.380). This was similar to the study in Kano and Abuja.^19^, ^24^ Other maternal complications such as puerperal sepsis, post-partum haemorrhage, surgical site infection, caesarean hysterectomy were found more amongst the emergency caesarean section patients. More so, prolonged hospital stay was commoner among patients that had emergency caesarean section and was statistically significant (x^2^=5.960, df=1, p=0.015). Maternal deaths were found only in those who has emergency caesarean section although not statistically significant (x^2^=2.197, df=1, p=0.138).

## Conclusion

This study showed a c/s rate of 25.4% with repeat caesarean section and cephalopelvic disproportion being the most common indication for elective and emergency caesarean section respectively. Emergency caesarean section accounted for most of the cases and is associated with increased maternal and perinatal morbidity and mortality.

## Recommendations

It is thus recommended that efforts should be made towards reducing primary caesarean section and in addition to improve trial of vaginal delivery services. Also health education of the populace about the need for supervised pregnancy and delivery by skilled birth attendants. There should be a feedback mechanism between tertiary facilities and secondary as well as primary health centres, with emphasis on the need for early referral of patients.

## References

[1] Incerpi MH, Decherney AH, Nathan L, Goodwin TM, Laufer N (2013). Operative delivery. Current diagnosis and treatment obstetrics and gynaecology.

[2] Hiralar K (2015). Operative delivery. DC Dutta's Textbook of Obstetrics.

[3] Cunningham FG, Leveno KJ, Bloom SL, Dashe JS, Hoffman BL, Casey BM, Spong CY (2018). Caesarean delivery and peripartum hysterectomy. Williams Obstetrics.

[4] Ikechebelu JI, Mbamara SU, Afuba AN (2010). Vaginal birth after one caesarean section: A review of the practice at Nnewi, Southeast Nigeria. J Med Med Sci.

[5] Strom S (2013). Rates, Trends, and Determinants of Cesarean Section Deliveries in El Salvador: 1998 to 2008 (doctoral dissertation).

[6] Betrán AP, Ye J, Moller A-B (2016). The increasing trend in caesarean section rates: global, regional and national estimates: 1990–2014. PLoS One.

[7] Onoh RC, Eze JN, Ezeonu PO, Lawani LO, Iyoke CA, Nkwo PO (2015). A 10-year appraisal of cesarean delivery and the associated fetal and maternal outcomes at a teaching hospital in southeast Nigeria. Int J Womens Health.

[8] Eleje GU, Udigwe G, Akabuike J, Eke A (2010). The Rate of Caesarean Section in Nnewi, Nigeria : A 10-year. Afrimedic J.

[9] Adekanle D, Adeyemi A, Fasanu A (2013). Caesarean section at a tertiary institution in Southwestern Nigeria—A 6-year audit. Open J Obstet Gynecol.

[10] Nwobodo EI, Isah AY, Panti A (2011). Elective caesarean section in a tertiary hospital in Sokoto, north western Nigeria. Niger Med J.

[11] Ezugwu EC, Iyoke CA, Iloghalu IE, Ugwu EO, Okeke TC, Ekwuazi KE (2017). Cesarean section rate and its outcome in a tertiary hospital in Enugu, South-east Nigeria. Int J Med Health Dev.

[12] John CO, Alegbeleye JO (2017). Caesarean Delivery at a Teaching Hospital, South-South Nigeria: A Five-Year Review. International Journal of Tropical Disease & Health.

[13] Igberase GO, Ebeigbe PN, Andrew BO (2009). High caesarean section rate: a ten year experience in a tertiary hospital in the Niger Delta, Nigeria. Niger J Clin Pract.

[14] Torloni MR, Betran AP, Souza JP, Widmer M, Allen T, Gulmezoglu M (2011). Classifications for cesarean section: A systematic review. PLoS One.

[15] Miri FL, Abbasi SM (2012). Caesarean section change trends in Iran and some demographic factors associated with them in the past three decades. J Fasa Univ Med Sci.

[16] Grivell MR, Dodd JM (2011). Short and long term outcomes after caesarean section. Expert Rev of Obtet Gynaecol.

[17] Okeke TC, Onah N, Ikeako LC, Ezenyeaku CC, Nwogu Ikojo C (2013). Maternal and fetal outcome of elective caesarean section at 37 38 completed weeks of gestation in Enugu, Southeast Nigeria. Am J Clin Med Res.

[18] Prah James (2017). Caesarean section in a primary health facility in Ghana: Clinical indications and feto-maternal outcomes. Journal of Public Health in Africa.

[19] Isah AD, Adewole N, Zaman J (2018). A five-year survey of cesarean delivery at a Nigerian tertiary hospital. Trop J Obstet Gynaecol.

[20] Nnadi DC, Singh S, Ahmed Y, Siddique S, Bilal S (2016). Maternal and fetal outcomes following cesarean deliveries: A cross-sectional study in a tertiary health institution in North-Western Nigeria. Sahel Med J.

[21] Maanongun MT, Ornguze AA, Ojabo AO (2017). Indications and the materno-foetal outcome of caesarean section in a secondary health facility in obudu, southsouth Nigeria. Res Rep Gynaecol Obstet.

[22] (2013). Bull World Health Organ.

[23] WHO (2015). WHO Statement on Caesarean Section Rates.

[24] Abubakar I S, Rabiu A, Mohammed A D (2015). Magnitude and Pattern of Caesarean Sections in a Teaching Hospital, Northwest Nigeria: A 5 Year Analysis. Journal of Gynecology and Obstetrics.

[25] Ugwu EOV (2011). A Five-year Survey of Caesarean Delivery at a Nigerian Tertiary Hospital. Ann Med Health Sci Res Jan.

[26] Information Centre (2009). Method of delivery, 1980 to 2007-8. NHS maternity statistics, England: 2008-09.

